# Interprofessional Collaboration: Differentiating Netherton Syndrome and Atopic Dermatitis in an African American Infant

**DOI:** 10.7759/cureus.55905

**Published:** 2024-03-10

**Authors:** Erin G Park, Kylie Besly, Juan J Campos, Ahmed Rezk

**Affiliations:** 1 Medicine, Alabama College of Osteopathic Medicine, Panama City, USA; 2 Medicine, Alabama College of Osteopathic Medicine, Dothan, USA; 3 Pediatrics, Rainbow Pediatrics, Panama City, USA

**Keywords:** pediatric dermatology, african american pediatric eczema, chronic eczema, african american dermatology, african american infant, netherton syndrome

## Abstract

Netherton syndrome is a rare, autosomal recessive disorder that clinically presents with a triad of congenital ichthyosiform erythroderma, hair shaft abnormalities, and immune dysregulation, which is confirmed with genetic testing for mutations in the serine protease inhibitor Kazal-type 5 (SPINK5) gene. This diagnosis was included in our differential due to the patient's recurring and unimproving rash with desquamating skin. While eczema was included in our differential diagnoses, the patient's systemic symptoms, including failure to thrive, prompted our team to consider other diagnoses. This patient endured numerous treatments and diagnostic tests to eliminate underlying immunodeficiencies and autoinflammatory diseases. In this case report, we present a two-month-old male who was originally brought into the outpatient pediatric clinic for severe eczema, periorbital swelling, and failure to thrive. The patient returned with a continuing exudative rash after amoxicillin suspension treatment and was ultimately hospitalized for IV antibiotic treatment. The patient was then transferred to multiple hospitals for treatment and final diagnosis of severe seborrheic dermatitis and atopic dermatitis. Multiple inpatient hospitals and outpatient clinics had to collaborate and communicate effectively to reach a diagnosis. The diagnosis for this patient was found after a true display of the value of interdisciplinary collaboration as several outpatient clinics and inpatient hospitals worked together for this outcome.

## Introduction

Netherton syndrome is a complex disorder that affects various organ systems, and it is characterized by congenital ichthyosiform erythroderma, hair shaft irregularities, and immune dysregulation [1]. While the specific pathophysiology of this condition is not fully understood, a loss-of-function mutation in the serine protease inhibitor Kazal-type 5 (SPINK5) gene, encoding a serine protease inhibitor lymphoepithelial Kazal-type-related inhibitor (LEKTI), is thought to contribute to progressive inflammation and skin deterioration [1,2]. Part of this condition's presentation involves high serum immunoglobulin E (IgE), which may present similarly to atopic dermatitis, also known as eczema [1].

Eczema is a common inflammatory skin disease characterized by a pruritic rash and dry skin, and it has an 80% incidence in young individuals, with improvement in symptoms by adolescence in 60% of this same group [3]. These lesions typically develop on the cheeks and lateral aspects of limbs in infants as well as flexural areas, but they can also develop in other skin regions [4]. There are two presentations found in children from birth to two years of age, one characterized by transient progression and the other characterized by persistent progression [5]. Primary management of this condition should involve consistent moisturizing, but if the inflammation continues to progress, there is an increased risk for bacterial infections; topical anti-inflammatory medications such as corticosteroids and calcineurin inhibitors have been noted to prevent these infections [6].

Our case study presents a two-month-old male who was initially diagnosed and treated for severe eczema in an outpatient setting. However, the patient returned with infectious complications and needed to receive intravenous antibiotics in an inpatient setting. This article will focus on the management of an infant with complicated eczema and its distinction with Netherton syndrome.

## Case presentation

**Figure 1:**
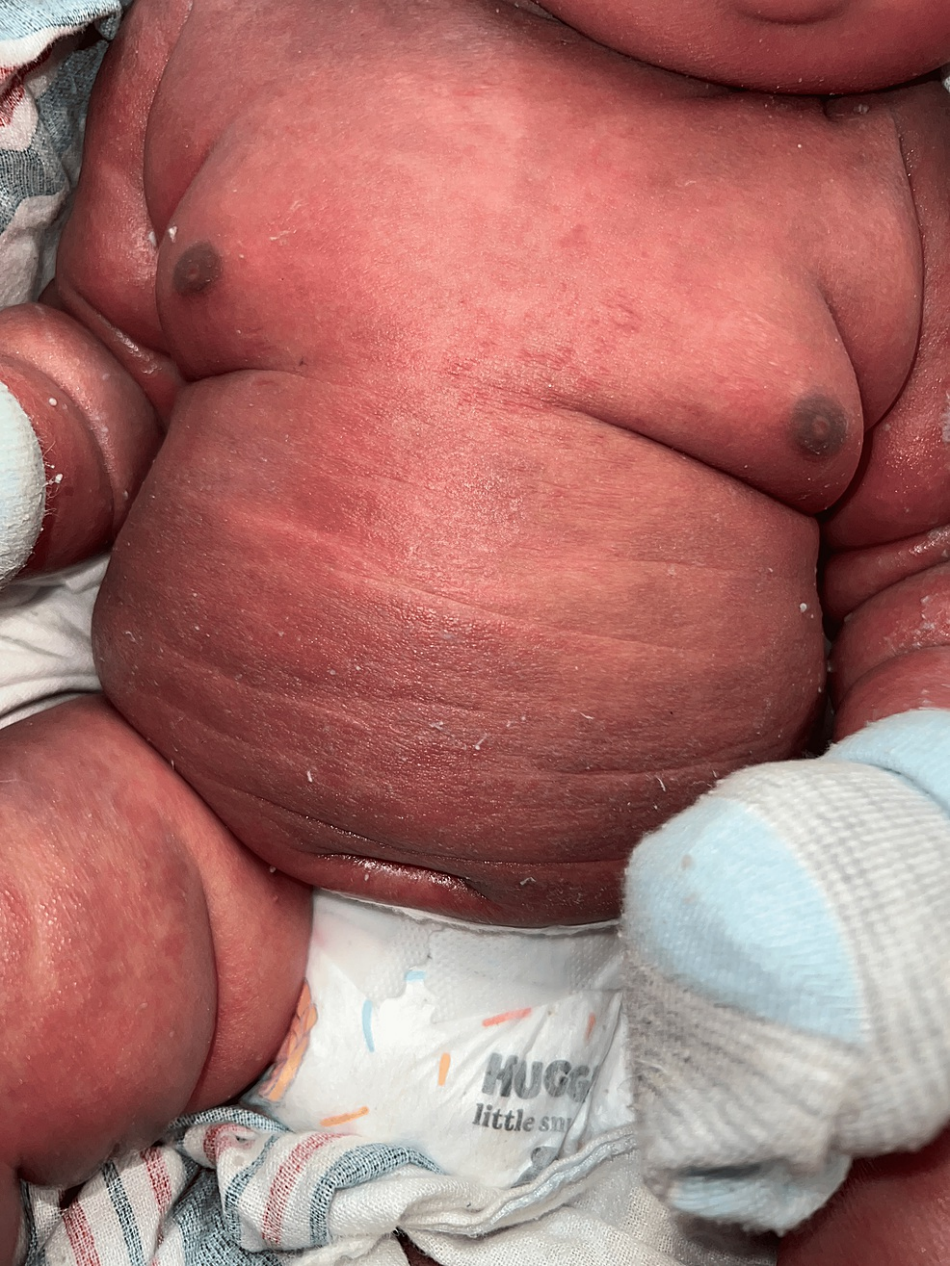
The patient's torso delineating nonbullous ichthyosiform erythroderma

**Figure 2:**
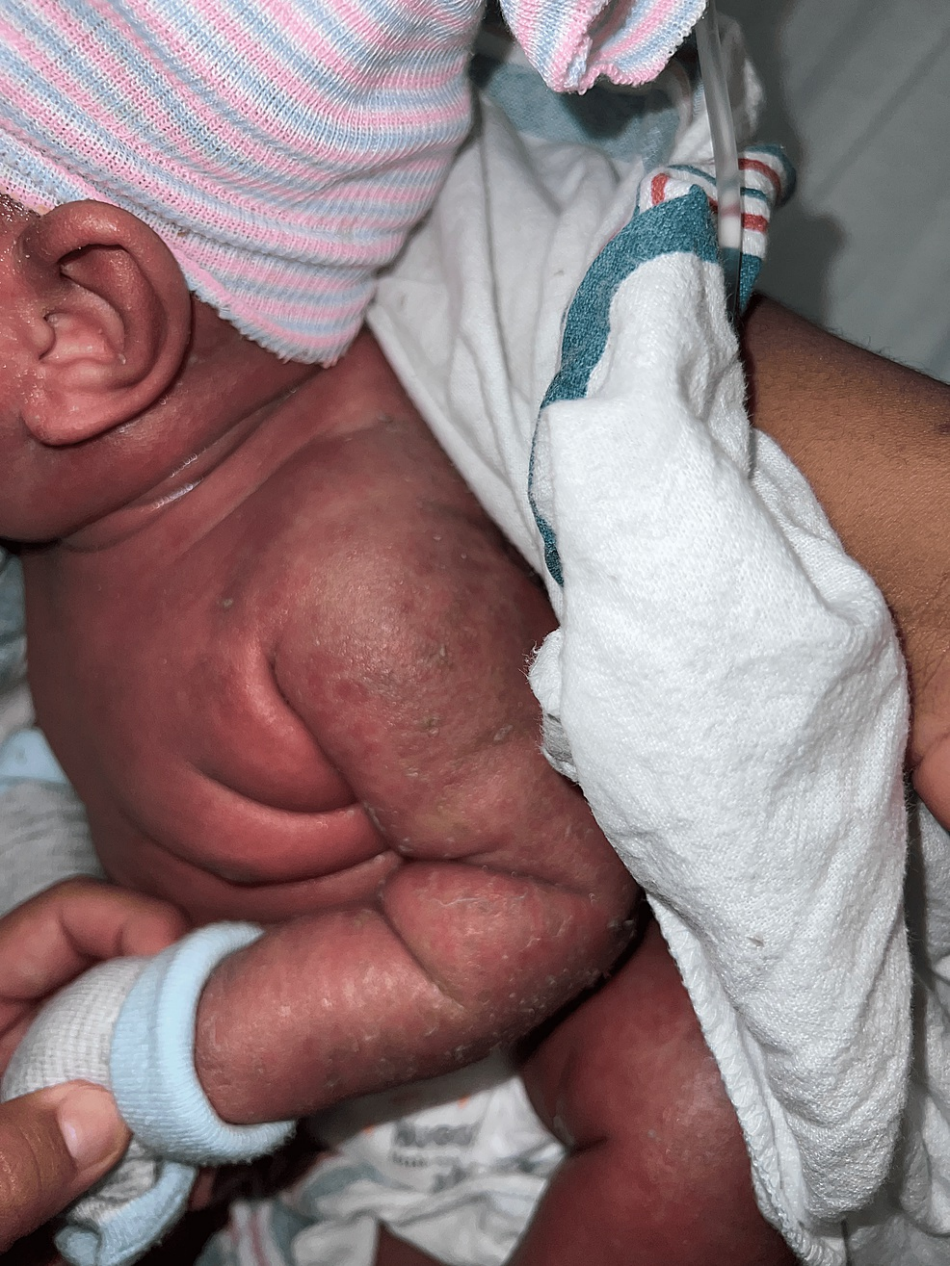
The patient's left upper extremity delineating nonbullous ichthyosiform erythroderma

**Figure 3:**
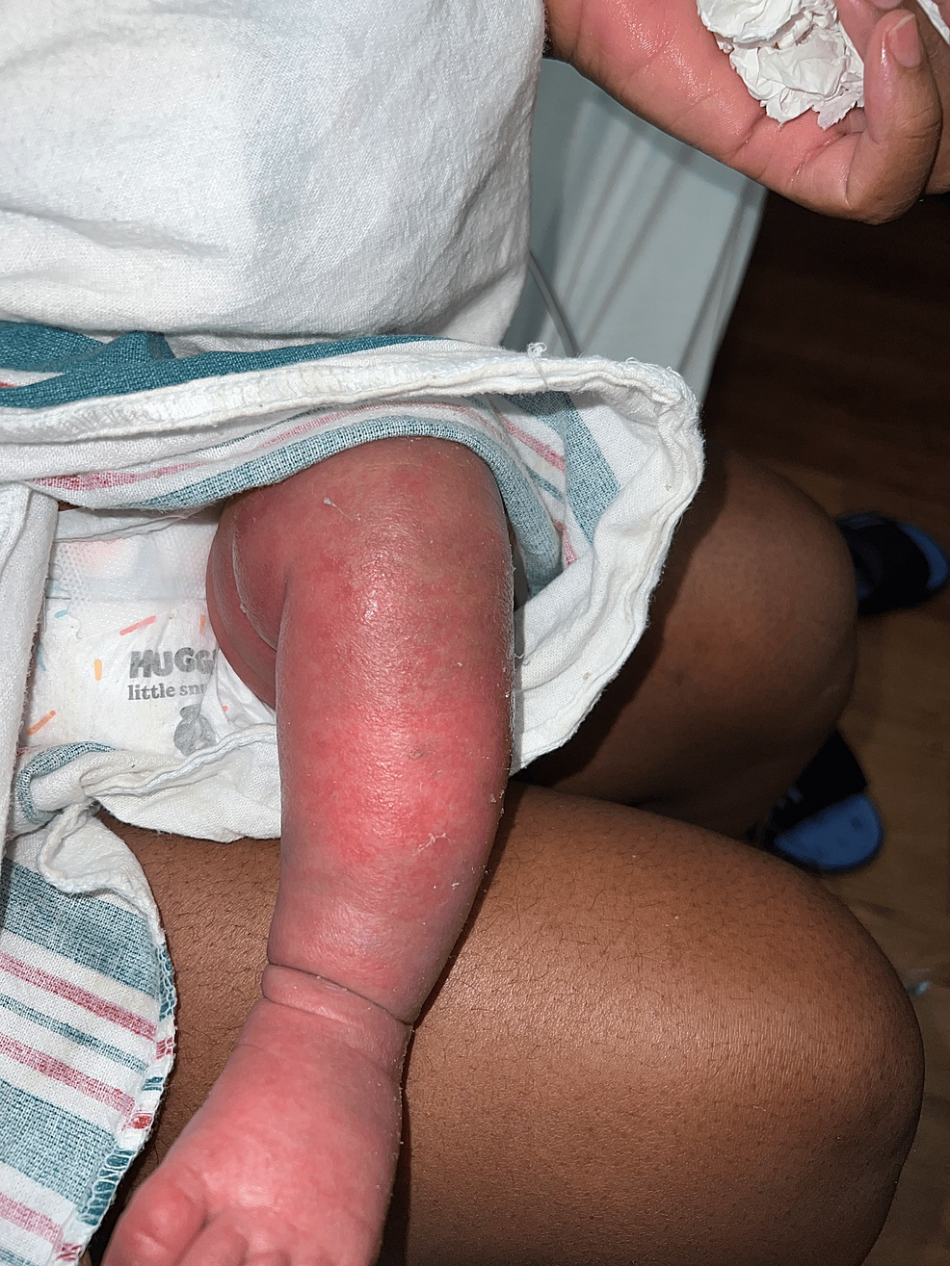
The patient's left lower extremity delineating nonbullous ichthyosiform erythroderma

**Figure 4:**
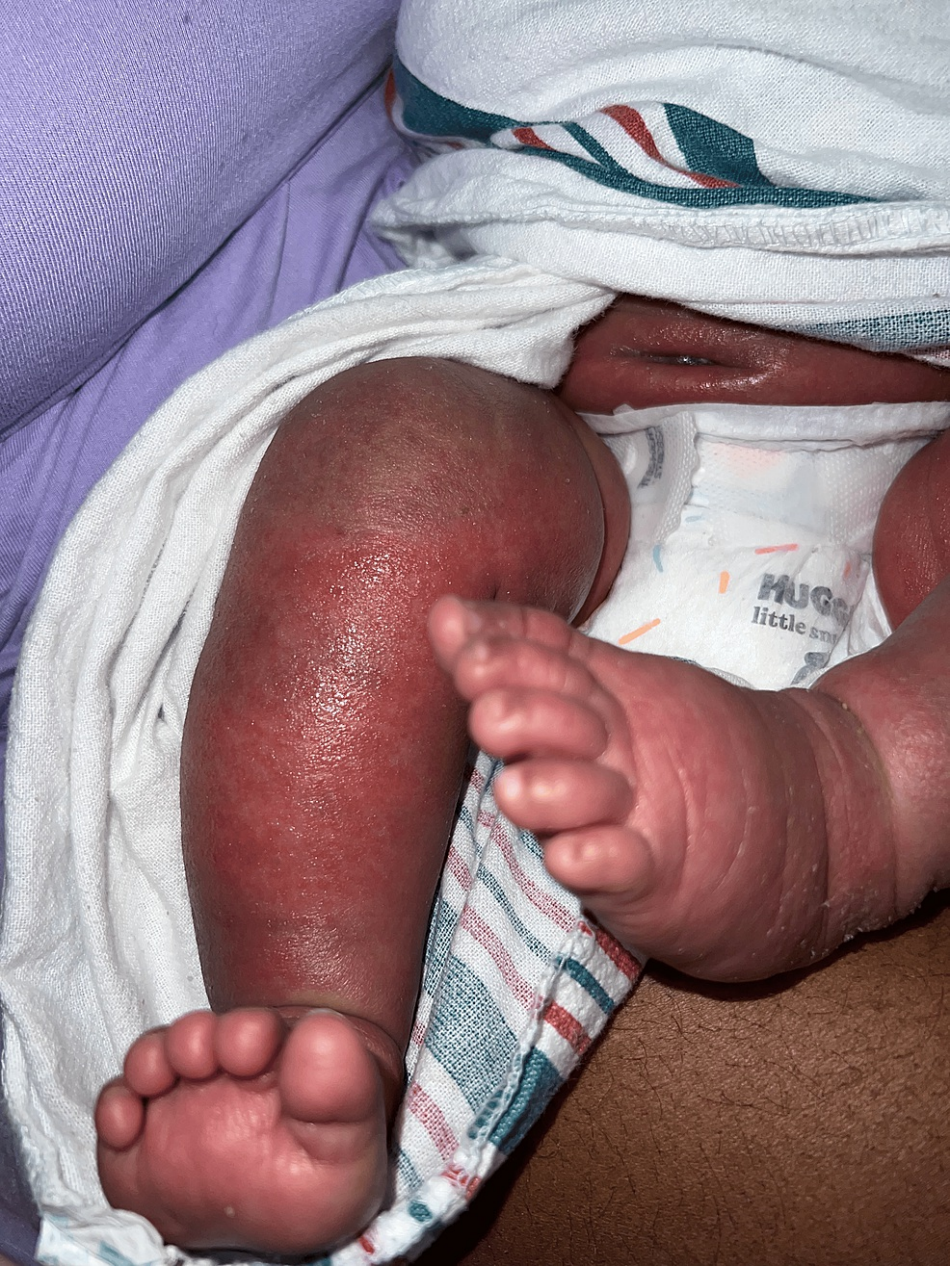
The patient's lower extremities delineating nonbullous ichthyosiform erythroderma

The two-month-old patient first presented at an outpatient clinic with bilateral upper and lower extremity and periorbital swelling with nonbullous ichthyosiform erythroderma. The patient was born full-term with no complications and was developing well until around one month of age. The patient's mother noticed a small rash on the patient's face around one month of age and attributed it to eczema. The pediatrician treated the patient's skin condition as eczema and encouraged the patient to continue aggressive moisturizing with over-the-counter lotions that included petroleum jelly and skin protectants that were approved for pediatric eczema. After one week, the patient returned to the outpatient clinic and did not show improvement. He continued to have redness and peeling skin, as seen in Figures 1-4.

**Figure 5:**
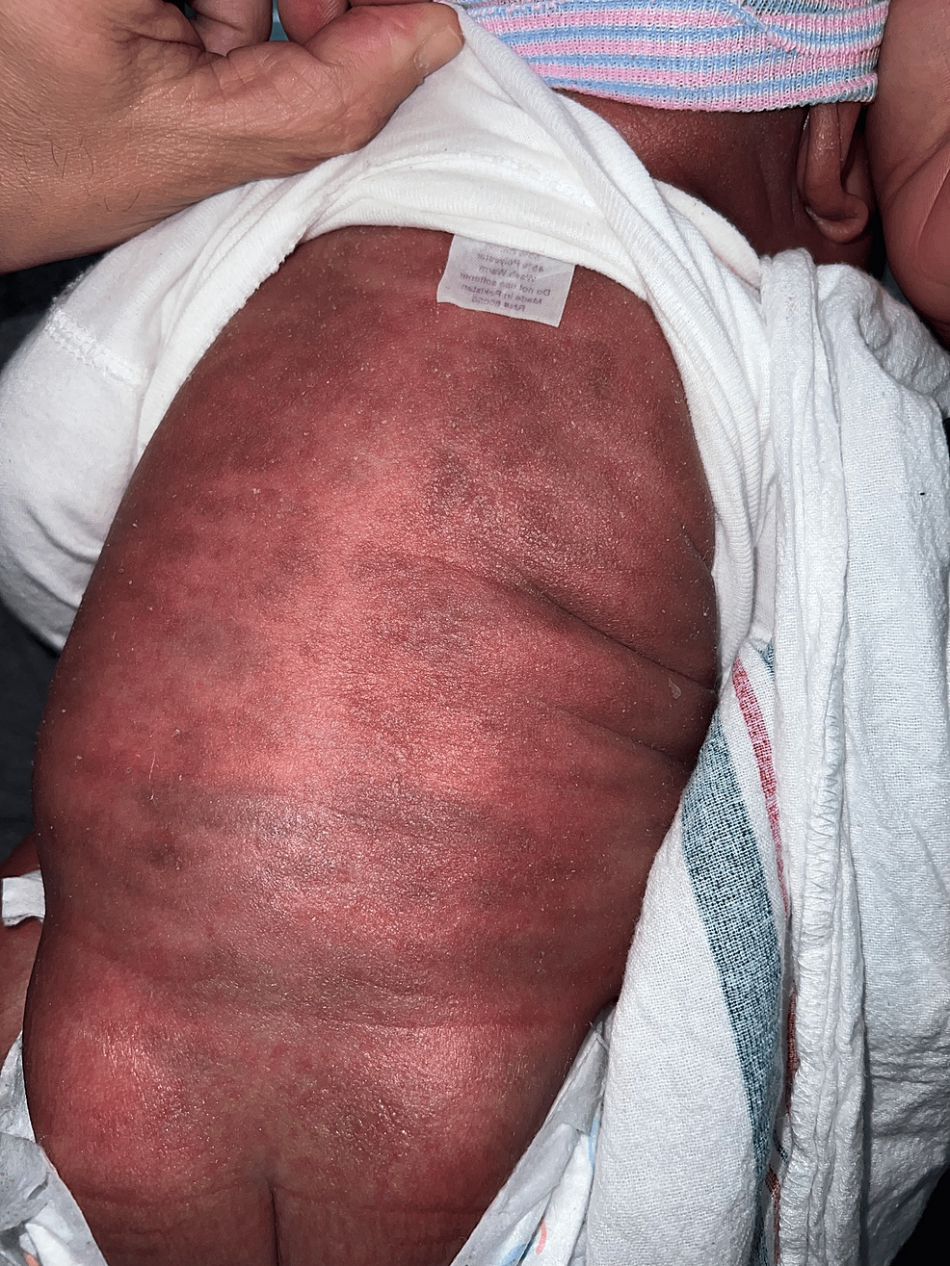
Slight improvement seen on the patient's back after four days of IV clindamycin, ceftriaxone, mupirocin (Bactroban) ointment, and nystatin ointment

**Figure 6:**
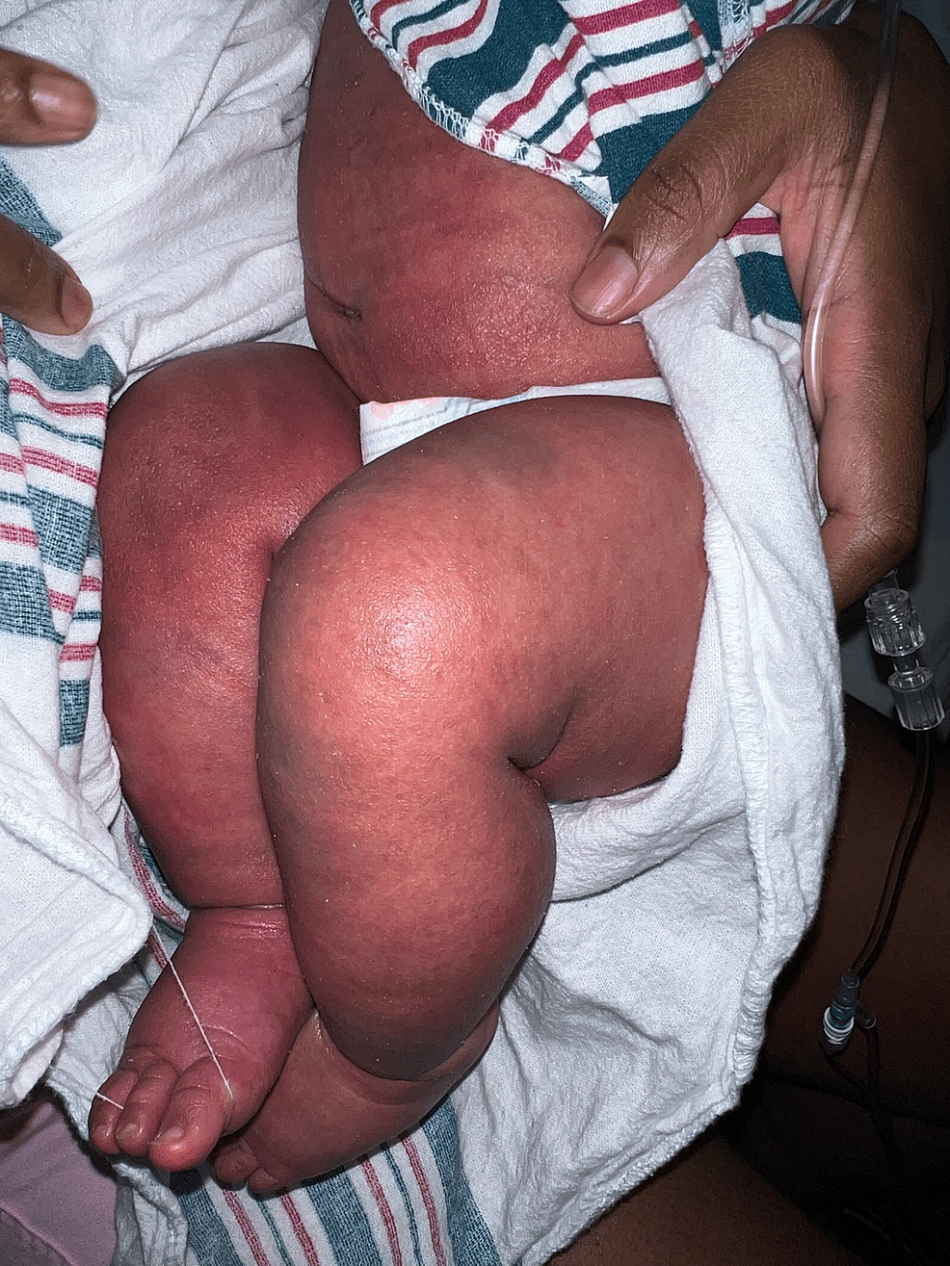
Slight improvement seen on the patient's lower extremities after four days of IV clindamycin, ceftriaxone, mupirocin (Bactroban) ointment, and nystatin ointment

**Figure 7:**
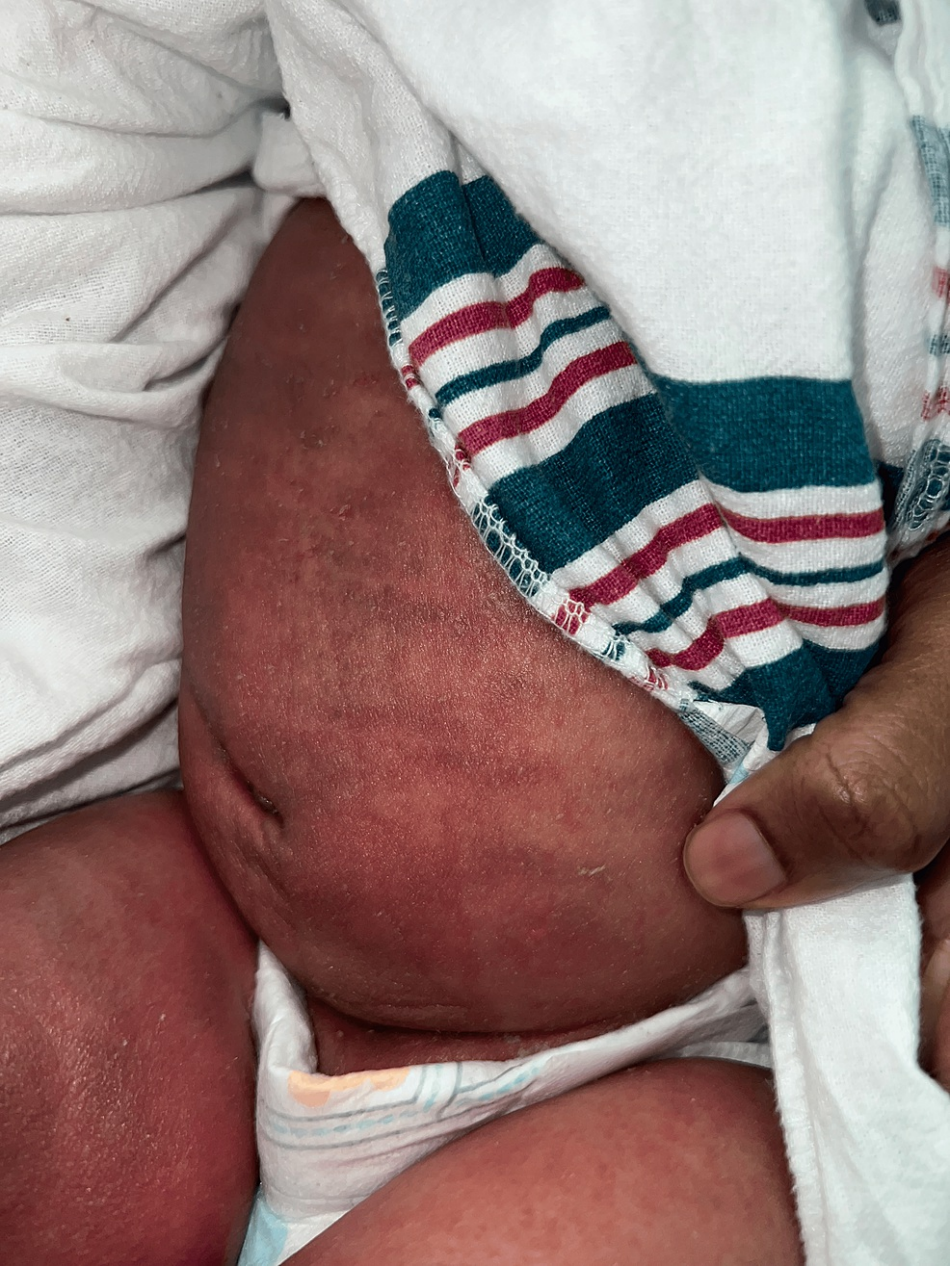
Slight improvement seen on the patient's abdomen after four days of IV clindamycin, ceftriaxone, mupirocin (Bactroban) ointment, and nystatin ointment.

The patient also showed a failure to thrive despite the addition of fortifying formula samples from the outpatient clinic. The patient was immediately hospitalized for intravenous (IV) antibiotics and diagnostic tests (complete blood count, comprehensive metabolic panel, coagulation profile, total protein and albumin/globulin ratio, immunoglobulins, and superficial skin culture). Skin culture confirmed methicillin-resistant *Staphylococcus aureus* (MRSA). Immunoglobulins were appropriately elevated with a MRSA infection. The patient received IV clindamycin, ceftriaxone, mupirocin (Bactroban) ointment, and nystatin ointment. The mother reported the patient's skin improving with IV clindamycin therapy (Figures 5-7), and the patient was discharged.

However, one week later, the patient returned to the outpatient clinic with a worsening rash, hair loss, and poor oral feeding. The patient was immediately admitted. During this admission, the patient was treated with IV vancomycin and cefepime. A nasogastric tube was also started to alleviate the patient's failure to thrive. The ordered coagulation profile showed abnormalities, as depicted in Table 1. The patient was also noted to have hypoalbuminemia, increased IgE, and transaminitis. Due to these lab values and the need for a higher level of care, the patient was then transferred to a larger pediatric hospital. Differential diagnoses included Netherton syndrome, Staphylococcal scalded skin syndrome, chronic eczema, and other less common causes of ichthyosiform erythroderma.

**Table 1:** Patient's coagulation profile of prothrombin time test (PT), partial thromboplastin time test (PTT), and international normalized ratio (INR)

	Patient's values	Normal values
PT	33.2 seconds	11-15 seconds
aPTT	61 seconds	25-40 seconds
INR	2.8	<1.1

The primary findings of an exon sequencing genetic test showed to be "heterozygous for a variant of uncertain significance in ABCC6", and was noted to be heterozygous in the ABCC6 gene for a sequence variant defined as c.2342C>T. This variant is predicted to result in the amino acid substitution p.Ala781Val. The clinical significance of this genetic variant was uncertain because of the absence of conclusive functional and genetic evidence. The patient was discharged and transferred to an acute care facility for specialized dermatologic treatment.

The pediatric dermatologist at the acute care facility changed the treatment to add topical triamcinolone (body), hydrocortisone (face), and ketoconazole (scalp). This treatment was deemed successful when the patient's skin began to clear. Pediatric endocrinology was consulted due to the patient developing adrenal suppression with steroid use. Dermatology stressed the importance of continuing topical treatment due to the patient's relapse when removing steroids. The patient was treated with a stress dose of oral hydrocortisone, and the parents were told to continue follow-up with outpatient pediatrics to monitor electrolytes and cortisol levels. Dermatology confirmed the diagnosis of seborrheic dermatitis and atopic dermatitis. There was a low suspicion for Netherton syndrome due to the positive response to topical steroids, the normal hair growth before hospitalization, and the genetic testing that did not show a SPINK5 mutation. A family history of the infant included eczema in both the mother and the father, strengthening the diagnosis of seborrheic dermatitis and atopic dermatitis. 

## Discussion

Skin turnover is a necessary biological process. In a nonmutated individual, this is in part mediated by serine protease inhibitor of Kazal type 5 (SPINK5), lymphoepithelial Kazal type inhibitor (LEKTI), and kallikrein 5 (KLK5). SPINK5 encodes for LEKTI, which, in a pH-dependent nature, will inhibit KLK5, a protease responsible for the recruitment of inflammatory mediators. In the natural process, LEKTI is activated by a neutral environment, like that of the deep epidermis, thereby inhibiting KLK5 and keeping the layer intact. This contrasts with acidic environments, like the superficial epidermis, in which LEKTI is inhibited, allowing KLK5 to activate and mediate the inflammatory process, initiating the sloughing of the superficial epidermis. It is this balance that allows for the regeneration of skin [7].

When this process is defective, as seen in Netherton syndrome (NS) and chronic eczema (CE), there is increased turnover of skin at deeper layers of the epidermis. Both NS and CE have a mutation in SPINK5 that causes a deficiency in LEKTI and subsequent overexpression of KLK5, which leads to a premature breakdown of corneodesmosomal components of the epidermis, disrupting the natural skin barrier [7]. This disruption releases a host of inflammatory cytokines, one of which is thymic stromal lymphopoietin (TSLP). TSLP activates Langerhans cells, which then activate Th2 cells to induce immunoglobulin class switching to IgE and the consequent recruitment of eosinophils. In both CE and NS, increased IgE and eosinophilia are noted by way of this process. In contrast, however, to CE, NS has a variety of other inflammatory cytokines recruited, leading to further breakdown in the skin barrier and increasing the risk for recurrent infection [8].

The two-month-old patient discussed in this case presented with cutaneous and laboratory findings common between both NS and CE. The diagnostic criteria for NS are allergic disease, like chronic eczema, plus at least one of the following: trichorrhexis invaginata ("bamboo hair"), family history of NS in a sibling, and identification of SPINK5 gene mutation. This patient was positive for allergic disease, as evidenced by the patient's cutaneous manifestations and elevated IgE and eosinophilia [8]. However, he did not have characteristic hair findings or a SPINK5 mutation, making a diagnosis of chronic eczema more likely.

Treatment for this patient will likely be on a relapsing-remitting basis. The first line is topical corticosteroids, to which the patient responded well. If the disease worsens, the patient may need to consider the use of biological agents vs Janus kinase (JAK) inhibitors [9]. However, JAK inhibitors such as pimecrolimus 1% cream are approved for mild to moderate atopic dermatitis in children over the age of two years [9]. Prevention is also an important component of treatment. Although a meta-analysis concluded that emollient use does not prevent the occurrence of atopic dermatitis, it is beneficial for symptomatic treatment and to improve comfort for those with established eczema [10]. One study showed that emollient use decreased incidence in a year by 14% [11]. Vitamin D supplementation is another way to prevent CE, especially that which results from the winter months [12]. These children are also at an increased risk for chronic and recurrent infections due to their defective skin barrier. Patients and their families should be aware that weeping, crusting, and pustules are red-flag symptoms of infection [9]. Despite the relapsing-remitting nature of this disease course and its increased incidence of infection, CE is a relatively common disease that can be followed with outpatient pediatrics. The child studied in this case had particularly severe CE, so close follow-up is recommended.

## Conclusions

Netherton syndrome is a rare pediatric condition that is diagnosed based on genetic testing and the clinical triad of congenital ichthyosiform erythroderma, hair shaft abnormalities, and immune dysregulation. Due to the patient's appropriate response to steroids, with a lack of typical features and an inconclusive genetic study, he was diagnosed as a case of chronic eczema. Since his case was severe, he was advised for regular follow-up in order to monitor the levels of steroids and cortisol along with their long-term consequences.
